# A Rare Case of RNRVAS Termination and Re‐Initiation Visualized on a 12‐Lead ECG

**DOI:** 10.1002/joa3.70270

**Published:** 2026-01-05

**Authors:** Tomoyoshi Morioku, Yasuyuki Egami, Yasuharu Matsunaga‐Lee, Masamichi Yano, Masami Nishino

**Affiliations:** ^1^ Clinical Engineering Technologist Osaka Rosai Hospital Osaka Japan; ^2^ Division of Cardiology Osaka Rosai Hospital Osaka Japan

**Keywords:** 12‐lead electrocardiogram, PVC response, repetitive non‐reentrant ventriculoatrial synchrony, ventricular intrinsic preference

## Abstract

This case illustrates both termination and re‐initiation of repetitive non‐reentrant ventriculoatrial synchrony (RNRVAS) visualized on a standard 12‐lead ECG. It highlights how pacemaker algorithms such as VIP and PVC response, together with abnormal atrial refractoriness, can trigger or terminate RNRVAS.

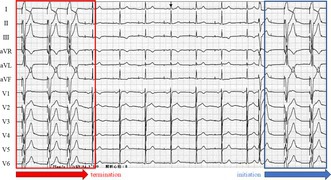

The diagnosis of pacemaker malfunction typically relies on telemetry interrogation. However, careful analysis of a 12‐lead electrocardiogram (ECG), combined with knowledge of pacemaker algorithms, enables accurate diagnosis without interrogation. A 76‐year‐old woman with sick sinus syndrome underwent dual‐chamber pacemaker implantation (Zephyr XL DR 5826, Abbott) six years earlier for symptomatic bradycardia. The pacemaker was programmed in DDD mode with the following settings: lower rate, 70 bpm; maximum tracking rate, 105 bpm; atrioventricular delay (AVD): paced/sensed, 250/225 ms; post‐ventricular atrial refractory period (PVARP), 275 ms; post‐ventricular atrial blanking (PVAB), 150 ms; ventricular intrinsic preference (VIP), ON with a 200‐ms AVD extension to encourage intrinsic conduction; and PVC response, ON.

She remained asymptomatic and continued follow‐up. During a routine visit, a 12‐lead ECG was recorded at rest (Figure 1). The ECG (25 mm/s, 10 mm/mV) revealed abrupt cessation and re‐initiation of ventricular pacing (VP), demonstrating repetitive non‐reentrant ventriculoatrial synchrony (RNRVAS), a rare pacemaker‐mediated arrhythmia (Figure 1B). VP stimulus (①) was followed by a retrograde atrial activation (② red circle), and the subsequent atrial pacing (AP) (③) occurred during the atrial refractory period, resulting in non‐capture. This sequence (VP → retrograde P wave → non‐capture of AP) repeated in a loop, fulfilling the diagnostic criteria for RNRVAS [1]. The ventriculoatrial (VA) conduction time (for both premature ventricular contraction [PVC] and VP) measured on the 12‐lead ECG was 160 ms. VA conduction was confirmed on the intracardiac electrogram during a pacemaker‐mediated tachycardia (PMT) event, where VP was followed by atrial sensing (AS). The VA conduction time was approximately 340 ms, exceeding the PVARP (275 ms) and resulting in AS recognition and PMT initiation (Figure 2). RNRVAS persisted until the first intrinsic ventricular beat, then terminated with VIP OFF. The VIP algorithm promotes intrinsic conduction by extending the AVD and switches between ON and OFF every 30 s with three cycles. VIP turns OFF when VP occurs for three consecutive beats during the extended AVD and turns ON when three consecutive VS events are detected either during the extended AVD or within the normal AVD. In this case, the sequence of VIP OFF and ON was as follows (Figure 3):

**FIGURE 1:**
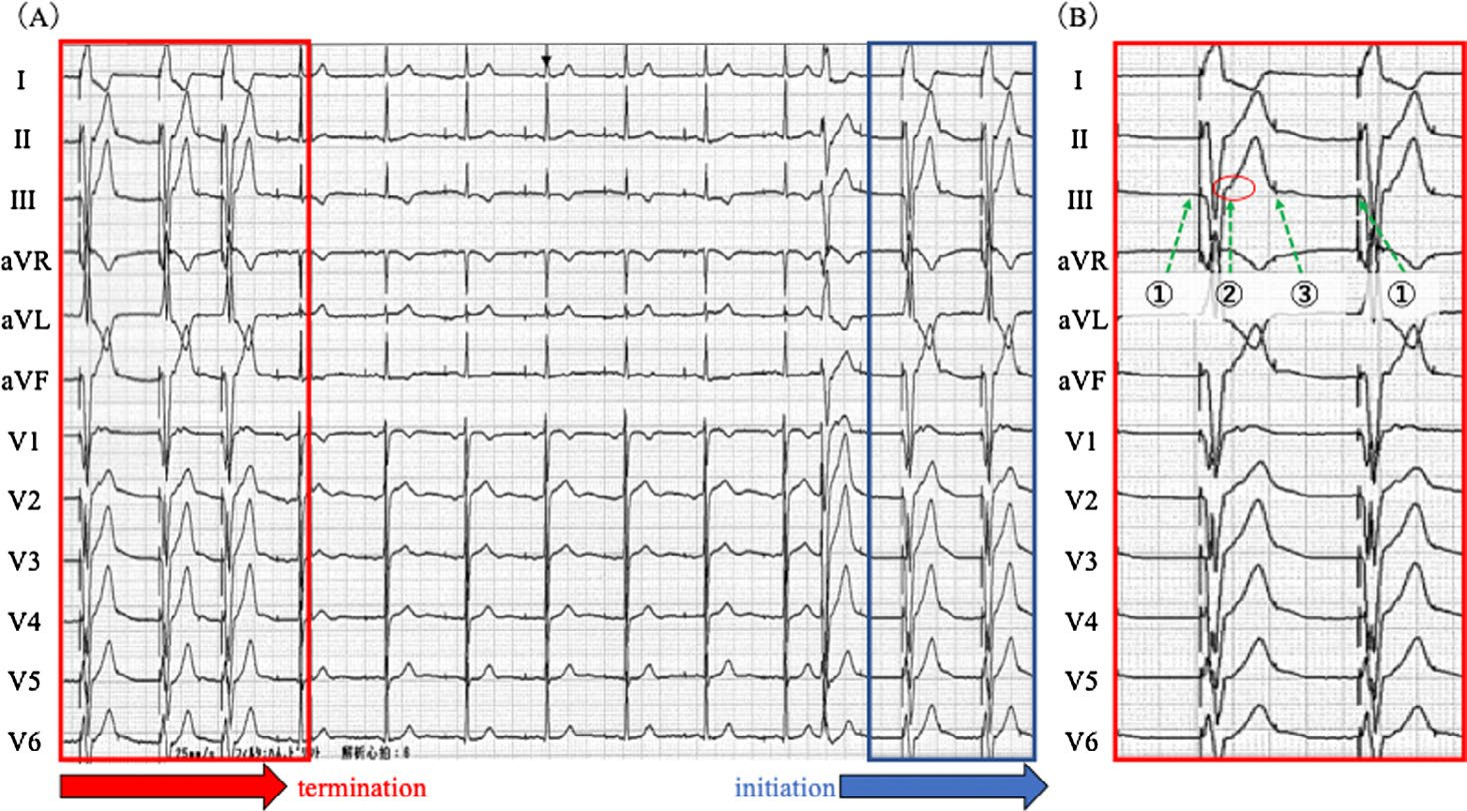
(A) A standard 12‐lead electrocardiogram showing abrupt cessation (red box) and re‐initiation (blue box) of ventricular pacing (VP), accompanied by a series of atypical rhythm changes. (B) A standard 12‐lead electrocardiogram is shown. Within the blue box, a characteristic pattern of RNRVAS is visualized: ① VP → ② retrograde P wave → ③ non‐captured AP due to atrial refractoriness, forming a repetitive loop. AP, atrial pacing; RNRVAS, repetitive non‐reentrant ventriculoatrial synchrony; VP, ventricular pacing.

**FIGURE 2:**
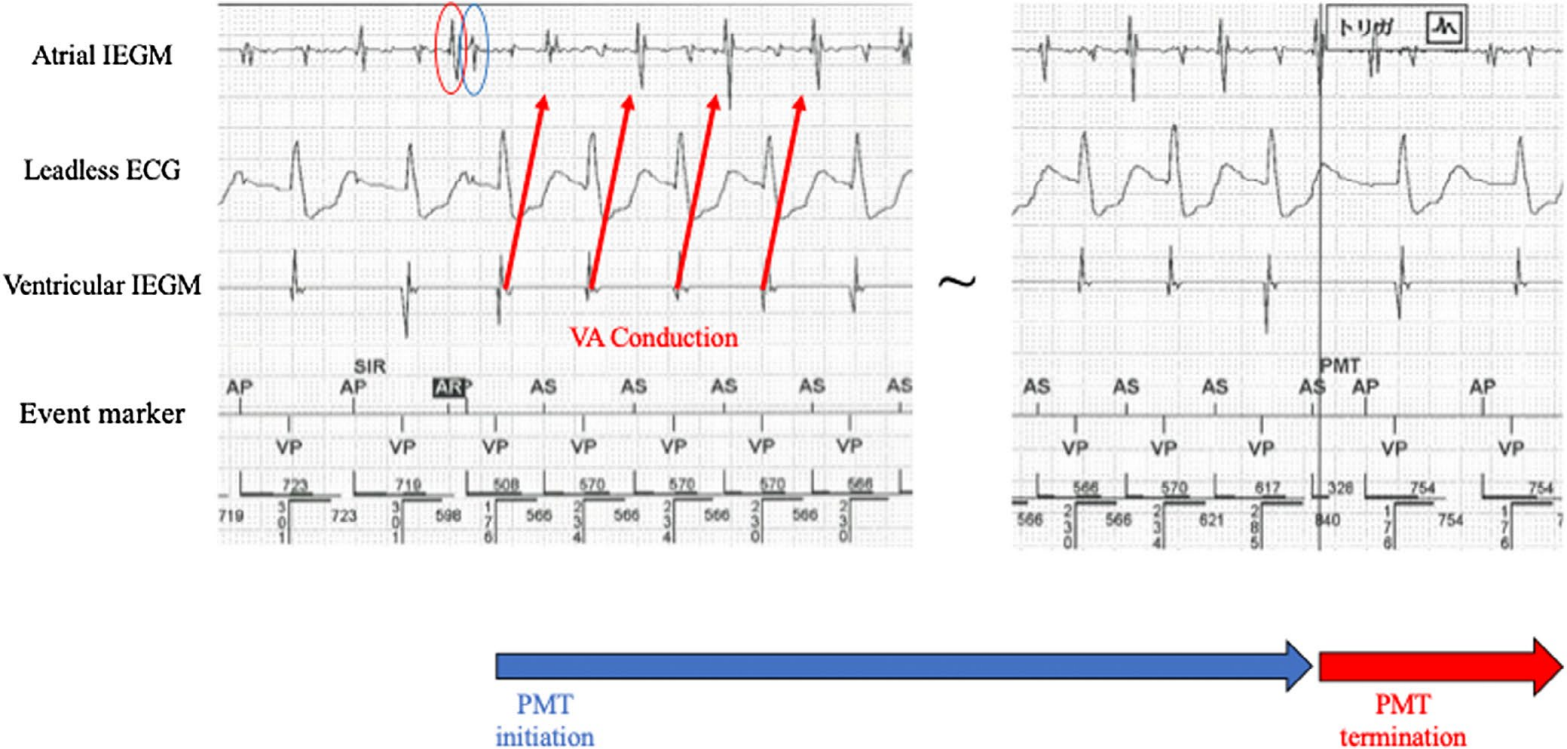
Atrial IEGM, leadless ECG, ventricular IEGM, and event marker channels are displayed. Immediately after an APC (red circle), AP (blue circle) was delivered during the atrial refractory period, followed by VP after the programmed AVD. Retrograde atrial activation was observed after VP, confirming the presence of VA conduction. This VA conduction triggered PMT, as indicated by the blue arrow. PMT was subsequently terminated by the device's PMT termination algorithm (red arrow). AP, atrial pacing; APC, atrial premature contraction; AR, atrial refractory; AS, atrial sensing; AVD, atrioventricular delay; ECG, electrocardiogram; IEGM, intracardiac electrogram; PMT, pacemaker‐mediated tachycardia; VA, ventriculoatrial; VP, ventricular pacing.

**FIGURE 3:**
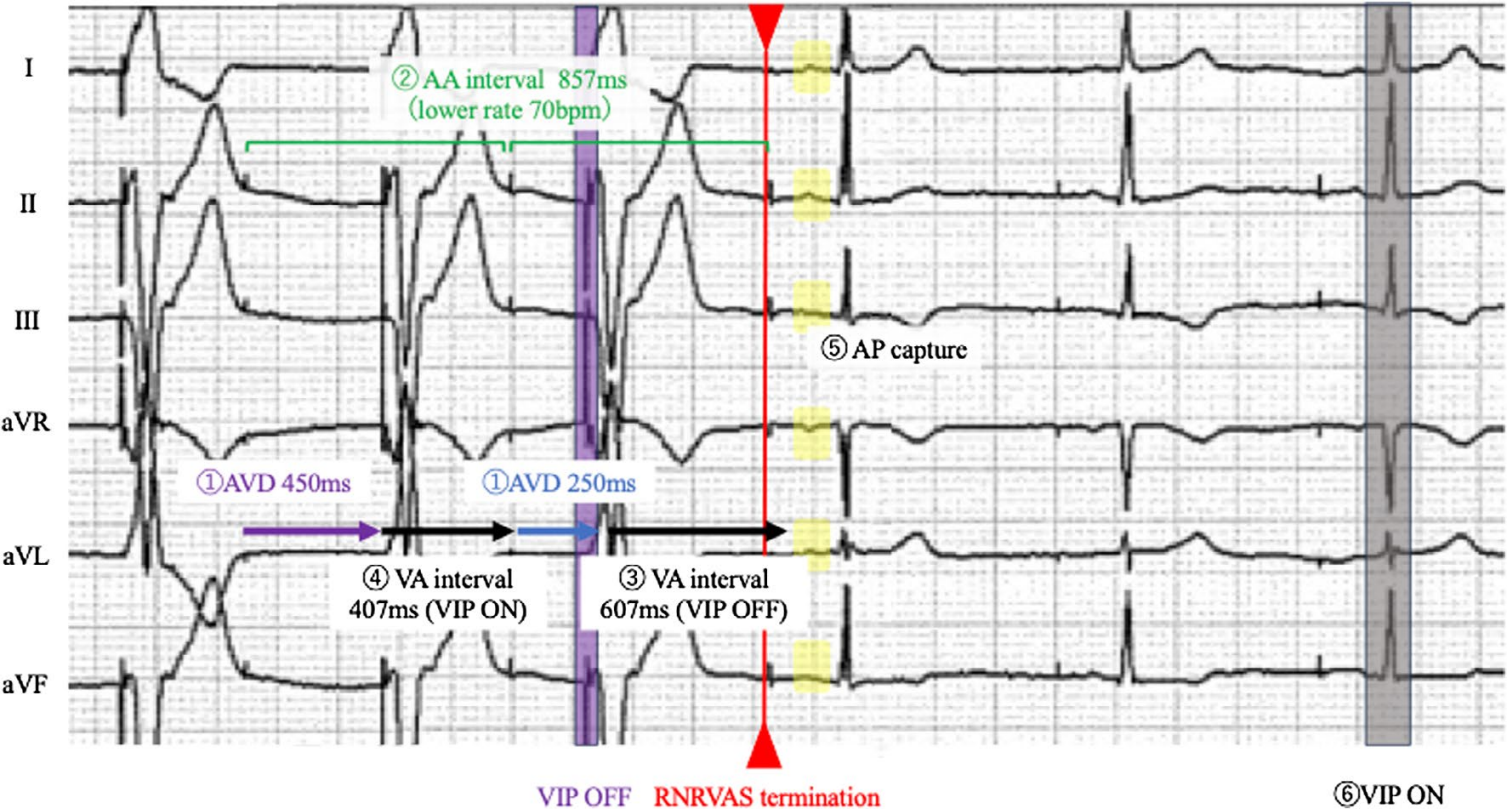
Limb leads extracted from the standard 12‐lead electrocardiogram are shown. Three consecutive VP events occurred during the extended AVD of 450 ms, resulting in VIP OFF (purple line). After VIP OFF, the AVD returned to its baseline value of 250 ms, and the VA interval was prolonged to 607 ms (857–250 = 607). In the previous beat, when VIP was ON, the VA interval was 407 ms (857–450 = 407). This prolonged VA interval allowed the AP stimulus to effectively capture the atrium (highlighted in yellow) beyond the physiologic refractory period caused by the preceding retrograde conduction, leading to the termination of RNRVAS (red line). Subsequently, VIP was reactivated (VIP ON) after three consecutive VS detections within the normal AVD (gray area). Abbreviations such as RNRVAS are defined in Figures 1A,B and 2. AA, atrial–atrial; PVARP, post‐ventricular atrial refractory period; VIP, ventricular intrinsic preference.

① Three consecutive VP events occurred during extended AVD of 450 ms.

② VIP turned OFF, and the AVD returned to its baseline value of 250 ms.

③ The VA interval was extended to 607 ms (857–250 = 607).

④ In the previous beat, when VIP was active, the VA interval was 407 ms (857–450 = 407).

⑤ This extended VA interval (③) allowed the AP stimulus to capture the atrium (highlighted in yellow) beyond the physiologic refractory period caused by the preceding retrograde conduction, resulting in RNRVAS termination. Thus, the transition from VIP ON to OFF status restored the baseline AVD, prolonged the VA interval, and interrupted the RNRVAS loop.

⑥ Subsequently, VIP was reactivated after three consecutive VS detections within the normal AVD.

At the time of RNRVAS initiation triggered by the PVC, the PVAB was 150 ms, the PVARP under PVC response was 480 ms, and the normal PVARP was 275 ms (Figure 4). As shown in Figure 4, a PVC (⑦ green box) was followed by a retrograde P wave (⑧ red circle). After the PVC, the pacemaker activated the PVC response algorithm designed to prevent PMT. The PVC response algorithm functions as follows: If a retrograde atrial event is sensed within the PVARP (extended to 480 ms), the device delivers an AP stimulus 330 ms after the atrial refractory (AR) event to restore atrioventricular synchrony and resume normal operation. Otherwise, if no atrial event is sensed within the PVARP, the device delivers AP after the programmed VA interval (Figure S1). In this case, the algorithm operated according to the first condition, delivering an AP stimulus 330 ms after AR detection (⑨ magenta arrows). However, because the atrium was still within its physiological refractory period, the AP resulted in non‐capture of AP (⑩ blue boxes). In this case, approximately 150 ms elapsed from the captured AP to the end of the atrial wave, suggesting abnormally prolonged atrial activation and refractory period likely leading to AP without atrial capture. Prolonged VA conduction, drug‐induced atrial refractoriness [2], and delayed atrial activation may contribute to RNRVAS development. This suggests that algorithms designed to prevent PMT may paradoxically trigger RNRVAS, highlighting the importance of understanding programming and atrial refractoriness.

**FIGURE 4:**
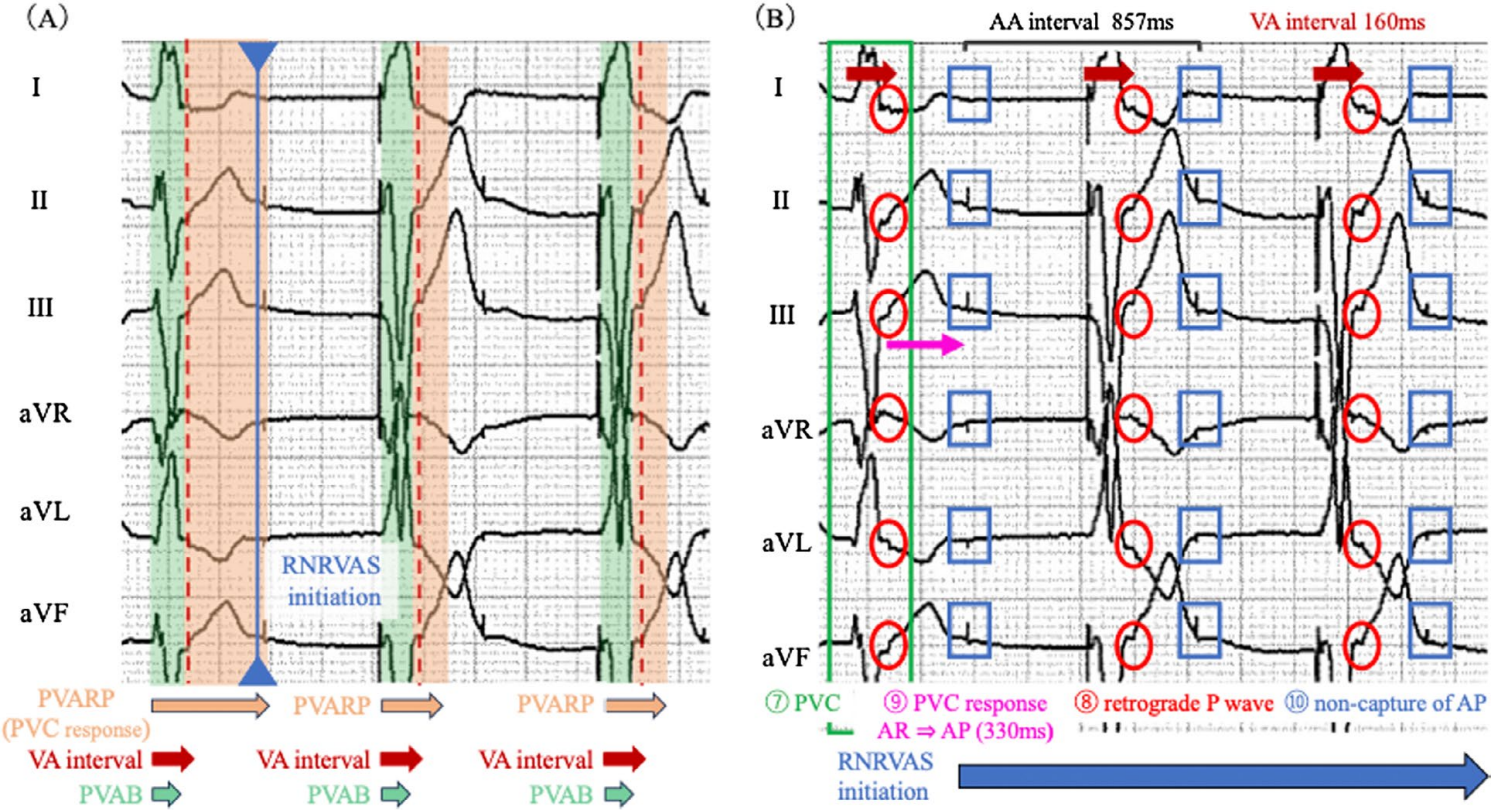
(A) Limb leads extracted from the standard 12‐lead electrocardiogram are shown. This figure illustrates the PVARP during PVC response, the normal PVARP, the PVAB, and the VA interval. Following a PVC, the PVC response algorithm was activated, resulting in an extended post‐ventricular atrial refractory period (PVARP, orange area). A retrograde P wave was sensed within this extended PVARP, and atrial pacing was delivered during the atrial refractory period (non‐capture). Consequently, RNRVAS was re‐initiated, as indicated by the blue line. (B) Limb leads extracted from the standard 12‐lead electrocardiogram illustrating the initiation of RNRVAS following a PVC (⑦, green box). The PVC is followed by a retrograde P wave (⑧, red circles), which is sensed during the PVARP. This triggers the device's PVC response algorithm, delivering AP 330 ms after AR sensing (⑨, magenta arrows). However, the atrium remains refractory at the time of pacing, resulting in non‐capture of AP stimuli (⑩, blue squares). This sequence repeats, initiating the RNRVAS cycle. The blue arrow highlights the continuous RNRVAS loop generated by this paradoxical algorithmic response. Abbreviations such as RNRVAS are defined in Figure 1. AA, atrial–atrial; AR, atrial refractory; PVAB, post‐ventricular atrial blanking; PVARP, post‐ventricular atrial refractory period; PVC, premature ventricular contraction; VA, ventriculoatrial.

This case represents a rare instance in which both termination and re‐initiation of RNRVAS were clearly documented on a standard 12‐lead ECG. Unlike reports using intracardiac electrograms or Holter monitoring [3], this case demonstrates the full RNRVAS sequence on surface ECG. To distinguish RNRVAS from other pacemaker‐mediated rhythms, understanding pacing dynamics and rate characteristics is essential. Among these, PMT is most representative. RNRVAS occurs near the lower pacing rate, whereas PMT appears at the upper tracking rate, where retrograde P waves are tracked as atrial sensing. Recognizing these differences allows differentiation using a 12‐lead ECG.

In this case, both pacing algorithms (VIP and PVC response) and the abnormally prolonged atrial activation with its refractory period may have contributed to RNRVAS termination and recurrence. Although triggered by a PVC, RNRVAS might also occur during ventricular pacing with an AVD plus VIP extension under atrioventricular block. Other manufacturers' platforms rely on post‐PVC PVARP extension without early atrial pacing, making this specific mechanism of RNRVAS less likely. These manufacturers‐specific differences underscore the need to tailor programming to the device and patient physiology. In addition, lowering the lower pacing rate from 70 to 60 bpm to avoid atrial pacing during the refractory period caused by retrograde conduction delayed the timing of atrial pacing, restored atrial capture, and markedly reduced RNRVAS episodes. In this case, RNRVAS was asymptomatic and incidentally detected. However, previous reports have shown that atrial pacing during the relative refractory period, also known as the vulnerable period, during RNRVAS can induce atrial arrhythmias [2], and that atrioventricular dyssynchrony may worsen heart failure [4]. Therefore, even asymptomatic RNRVAS warrants consideration of device reprogramming to prevent future arrhythmias or heart failure.

## Funding

The authors have nothing to report.

## Ethics Statement

The authors have nothing to report.

## Consent

The patient provided written informed consent for the ablation procedures and agreed to the publication of his case details and images in this report.

## Conflicts of Interest

The authors declare no conflicts of interest.

## Supporting information


**Figure S1:** joa370270‐sup‐0001‐FigureS1.zip.

## Data Availability

The data that support the findings of this study are available from the corresponding author upon reasonable request.
