# Supplementary material for: A Rare Case of RNRVAS Termination and Re‐Initiation Visualized on a 12‐Lead ECG
Source: J Arrhythm. 2026 Jan 5;42(1):e70270. doi: 10.1002/joa3.70270 (PMC12771595; doi:10.1002/joa3.70270)
Supplement: Supplementary file 1 — Figure S1: joa370270‐sup‐0001‐FigureS1.zip. [file JOA3-42-e70270-s001.zip › joa370270-sup-0002-Supinfo2@Supplementary file Figure Legend .docx]

**Supplementary figure.

Figure Legend**The PVC response algorithm functions differently depending on whether an atrial event (mainly due to retrograde conduction) occurs within the PVARP following a ventricular event. This algorithm has two operation patterns as follows:

**Pattern 1 (Left panel): Atrial event within PVARP.**When an atrial event is sensed within the PVARP, the device immediately terminates the refractory period and delivers an AP stimulus 330 ms after the detection of the AR event. This action allows the device to restore AV synchrony and return to normal operation. **Pattern 2 (Right panel): No atrial event within PVARP.**
If no atrial event is sensed within the PVARP (i.e., in the absence of retrograde conduction), the device delivers AP after the programmed VA interval and functions as a ventricular–ventricular counter. The schematic ECG was drawn at a standard speed of 25 mm/s and amplitude of 10 mm/mV.

In this case, with a lower rate limit of 70 bpm (basic cycle length of 857 ms), the device operated the ventriculoatrial counter calculated as “lower rate − paced AVD + VIP extension (857 − [250 + 200])” after PVC detection, delivering an AP approximately 407 ms later.On the other hand, the retrograde P wave after the PVC was conducted at 160 ms, and adding the PVC response interval of 330 ms gives 490 ms, resulting in a difference of about 83 ms. Therefore, we consider that the observed AP was due to the PVC response algorithm.
PVARP = post-ventricular atrial refractory period; AP = atrial pacing; AR = atrial refractory; AV = atrioventricular; PVC = premature ventricular contraction; VA = ventriculoatrial; VIP = Ventricular Intrinsic Preference; AVD = atrioventricular delay.
