# Supplementary figures and images for: A Rare Case of RNRVAS Termination and Re‐Initiation Visualized on a 12‐Lead ECG
Source: J Arrhythm. 2026 Jan 5;42(1):e70270. doi: 10.1002/joa3.70270 (PMC12771595; doi:10.1002/joa3.70270)

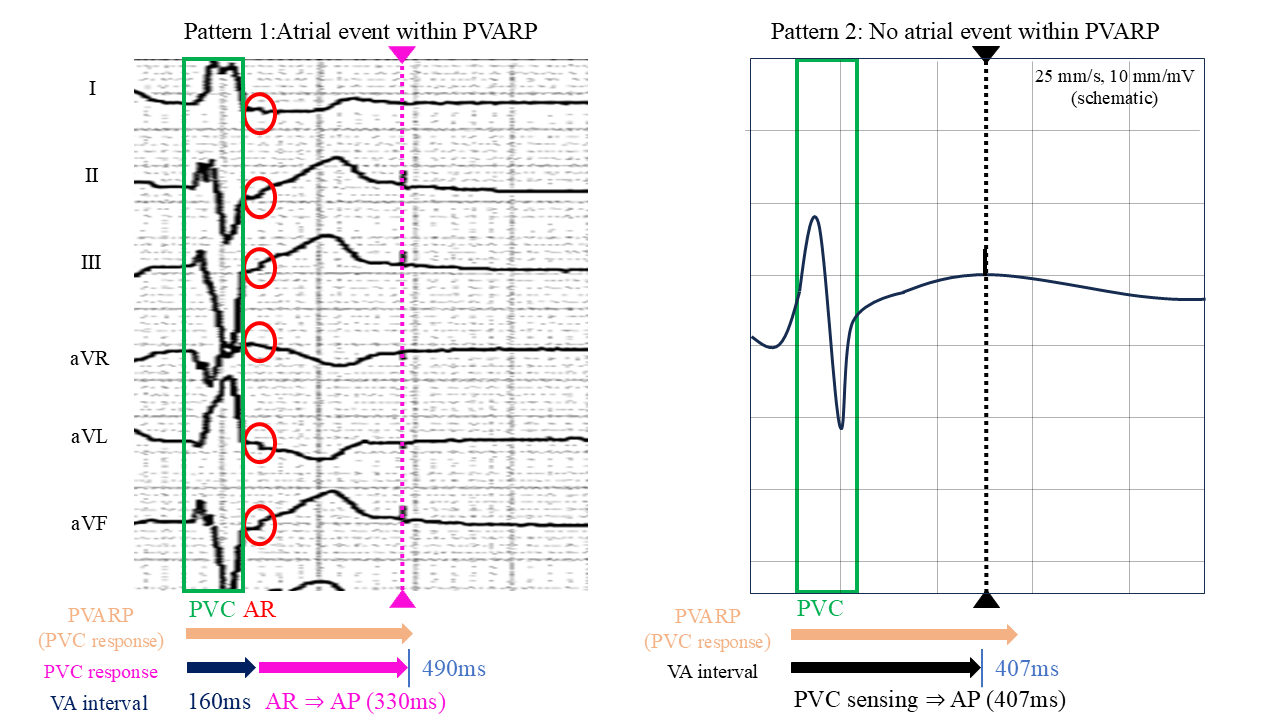

Supplement: Supplementary file 1 — Figure S1: joa370270‐sup‐0001‐FigureS1.zip. [file JOA3-42-e70270-s001.zip › joa370270-sup-0001-Supinfo1@Supplemental figure.tif]
